# Near-infrared imaging of head and neck squamous cell carcinoma using indocyanine green that targets the αvβ6 peptide

**DOI:** 10.1117/1.JBO.29.4.046002

**Published:** 2024-04-17

**Authors:** Yuan Yuan, Tengfei Fan, Jingbo Wang, Ying Yuan, Xiaofeng Tao

**Affiliations:** a Ninth People’s Hospital, Shanghai Jiao Tong University, School of Medicine, Department of Radiology, Shanghai, China; b Shanghai Ninth People’s Hospital, Shanghai Jiao Tong University School of Medicine, Department of Oral and Maxillofacial-Head Neck Oncology, Shanghai, China; c Shanghai Jiao Tong University, College of Stomatology, Shanghai, China; d The Second Xiangya Hospital of Central South University, Department of Oral and Maxillofacial Surgery, Changsha, China

**Keywords:** integrins, near-infrared imaging, head and neck squamous cell carcinoma, *α*v*β*6, indocyanine green, imaging

## Abstract

**Significance:**

Head and neck squamous cell carcinoma (HNSCC) has a particularly poor prognosis. Improving the surgical resection boundary, reducing local recurrence, and ultimately ameliorating the overall survival rate are the treatment goals.

**Aim:**

To obtain a complete surgical resection (R0 resection), we investigated the use of a fluorescent imaging probe that targets the integrin subtype αvβ6, which is upregulated in many kinds of epithelial cancer, using animal models.

**Approach:**

αvβ6 expression was detected using polymerase chain reaction (PCR) and immunoprotein blotting of human tissues for malignancy. Protein expression localization was observed. αvβ6 and epidermal growth factor receptor (EGFR) were quantified by PCR and immunoprotein blotting, and the biosafety of targeting the αvβ6 probe material was examined using Cell Counting Kit-8 assays. Indocyanine green (ICG) was used as a control to determine the localization of the probe at the cellular level. *In vivo* animal experiments were conducted through tail vein injections to evaluate the probe’s imaging effect and to confirm its targeting in tissue sections.

**Results:**

αvβ6 expression was higher than EGFR expression in HNSCC, and the probe showed good targeting in *in vivo* and *in vitro* experiments with a good safety profile.

**Conclusions:**

The ICG-αvβ6 peptide probe is an exceptional and sensitive imaging tool for HNSCC that can distinguish among tumor, normal, and inflammatory tissues.

## 1 Introduction

Head and neck squamous cell carcinoma (HNSCC) is the sixth most common cancer worldwide, with 890,000 new cases and 450,000 deaths as of 2018.^1^ The five-year relative survival rate for head and neck tumors ranged from 53% in 1970 to 67% in 2010.^2^ The survival rate for the early stages of the disease is 80%, but only 20% to 60% for the late stages.^3^ The survival rates have not improved significantly over the years due to various shortcomings. First, because of the insidious location of head and neck cancer, most patients are already in the progressive stage (tumor, node, and metastasis stage III-IV) at the initial diagnosis. Second, because of the deep anatomical location of the onset and infiltrating tissues of the lesion connecting the blood vessels and nerves, complete surgical resection (R0 resection) is difficult. Therefore, early diagnosis and improved surgical precision for resection are required to reduce recurrence and improve patient prognosis.

In early-stage patients, the lesions are usually limited; hence, surgical resection can be performed. In contrast, for patients with advanced disease, the treatment includes radiation and systemic therapy, followed by chemotherapy, immunotherapy, and simultaneous radiotherapy. The goal of surgery is to achieve complete resection with histological verification of the tumor-free margin; a negative margin is defined as >5  mm from the tumor margin.^4^ The gold standard at this stage is intraoperative frozen sectioning and postoperative determination of isolated specimens, but there are corresponding disadvantages; frozen sectioning is time-consuming and costly. Visual recognition and palpation to determine intraoperative borders have large errors. In addition, extensive excision can lead to physiological dysfunction.

Despite rapid advances in tumor imaging with X-rays, ultrasound, computed tomography, magnetic resonance imaging, and positron emission tomography, there are still practical difficulties in intraoperative imaging. Because of these limitations, new techniques are needed to monitor surgical margins in real time. Molecular imaging, first named by Weissleder in 1999, provides real-time visualization of tumors during intraoperative imaging-guided surgery.^5^ Probes for cancer diagnosis and staging are also being investigated for HNSCC for real-time intraoperative differentiation of cancer cells from adjacent normal tissues for molecular surgical margins to reduce cancer cells while preserving normal organ function. Recently, several near-infrared (NIR) probes have shown promising results in clinical trials. For example, PARP1-FL achieved high sensitivity and specificity in real-time imaging to discriminate between tumors and normal tissues in phase I clinical trials of oral cancer using smears.^6^ The probe EMI-137, which targets Met, has also shown good imaging results for isolated human tongue tumors.^7^

Indocyanine green (ICG) is a Food and Drug Administration-approved fluorescent agent. It has the following advantages: limited photostability, moderate fluorescence quantum yield, high plasma protein binding, and aggregation in aqueous solutions.^8^ A previous report documented the use of ICG in surgery,^9^ in which ICG signals were detected in 86% of tumors with good sensitivity but no specificity. Moreover, ICG is removed directly during pathological processing and cannot be used for *ex vivo* specimen analysis. Tumor-targeted NIR fluorescence probes are needed to map the margins of malignancy through the unique identification of biomarkers highly expressed in tumor cells. One article mentioned the need to develop bright, minimally toxic, tumor-specific NIR probes with smaller ligands as contrast agents for intraoperative imaging needs.^10^

Recently, integrins have received widespread attention as targets for tumor therapy. Integrins are a family of receptors that constitute the cell surface and promote cell adhesion to the extracellular matrix (ECM). Alpha and beta subunits form the heterodimer transmembrane receptor structure of integrins. There are 18 α and 8 β subunits, which contribute to 24 different integrins.^11^ Integrins perform two functions: both ligation, connecting cells to the ECM and receiving signals transduced from the ECM, and activation of signal transduction pathways, including signals related to cell growth, survival, division, and migration.^12^

Integrin αvβ6, the only integrin with epithelial specificity, is barely expressed in the normal epithelium but is highly expressed in various malignant epithelial tumors, including lung, ovarian, endometrial, oral, colorectal, skin, gastric, and idiopathic pulmonary fibrosis,^13^^–^^18^ making it a target for imaging. For example, it is expressed in up to 94.7% of head and neck squamous carcinomas.^19^ Integrin αvβ6 is expressed coherently during head and neck carcinogenesis. In one study, integrin αvβ6 was examined by immunohistochemistry (IHC) in oral leukoplakia, lichen planus, and squamous cell carcinoma, where it was expressed in precancerous lesions (41% in leukoplakia and 85% in lichen planus) but not during inflammation (proliferative or chronic). It was later found to be associated with malignant disease progression.^20^ αvβ6 is not expressed in the healthy epithelium but is upregulated during tissue remodeling processes, such as wound healing and carcinogenesis. It can regulate invasion, inhibit apoptosis, regulate proteases, and express activated transforming growth factor beta 1.^21^ The high expression of integrin αvβ6 in cancer is associated with low patient survival.^22^ Therefore, many radiopharmaceuticals targeting αvβ6 integrin on positron emission tomography have been developed recently, and some have been used in clinical trials,^23^^,^^24^ highlighting the clinical potential of αvβ6 in human tumor imaging and idiopathic pulmonary fibrosis in the lung. It has also been shown to accumulate specifically in tumors but not during inflammation.

In this experiment, we used the targeting peptide A20FMDV2 (NAVPNLRGDLQVLAQKVART), which targets αvβ6. This is a motif peptide derived from DLXXL of the foot-and-mouth disease (FMD) virus.^25^ It specifically targets αvβ6.^26^ We aimed to explore the role of ICG-αvβ6 peptide probes in HNSCC imaging. First, we investigated the expression of αvβ6 and epidermal growth factor receptor (EGFR) in human tissues with different degrees of malignancy. Subsequently, we compared the expression of αvβ6 and EGFR in normal and tumor cells. Cellular localization and imaging concentrations of the probes were determined in tumor cells. The safety of the probe was also determined using Cell Counting Kit-8 (CCK8). Finally, we performed *in vivo* imaging of the probe using an *in situ* tumor model and validated the targeting and sensitivity of the probe in tumor sections.

## 2 Methods

### 2.1 Imaging Agents

(ICG)-NAVPNLRGDLQVLAQKVART was purchased from China Peptides Co. Ltd. The amino acid peptide chain reaction was performed based on 2-chlorotrityl chloride resin with reference to the classical solid-phase peptide synthesis method. Kaiser reagent was used for monitoring during the reaction to ensure the linkage efficiency of amino acids and the efficiency of protecting group removal. All the reactions were performed at room temperature. ICG-COOH modification was performed using a condensation reagent for NHS activation, followed by the addition of a catalyst for linking to the N-terminus of the peptide. The resin and excess protecting groups were removed using trifluoroacetic acid, residual impurities were removed by reverse high-performance liquid chromatography, and the target material was collected and lyophilized to obtain the final product. The probe was prepared at 1  mg/mL concentration in 50% acetonitrile/50% $H_{2}O$.

### 2.2 Clinical Tissue and Hematoxylin-and-Eosin Staining

Formalin-fixed, paraffin-embedded biospecimens were collected from the Oral Surgery Department of the Ninth People’s Hospital. All diagnoses were made by pathologists through hematoxylin-and-eosin (HE)-stained sections. We analyzed specimens from four groups (normal [paraneoplastic tissue], n=6; benign [papilloma], n=6; precancerous [white spots], n=6; and malignant oral cancer tissue, n=6). All patients signed an informed consent form prior to surgery. The protocol number of the clinical research project of the Medical Ethics Committee was N0JBWKQA002.

### 2.3 Histology and Immunohistochemistry

Tissue sections were cut and immunohistochemically (IHC) stained for diagnosis. The tissues were divided into four groups according to the malignancy grade. Sections were dewaxed with xylene and alcohol, repaired with an antigen repair solution, and washed with phosphate-buffered saline (PBS). The samples were blocked after drawing circles around them; subsequently, primary antibodies for αvβ6 and EGFR were added dropwise at a temperature of 4°C overnight. The primary antibody was recovered, washed, and placed in the PBS solution. Then, color development was performed with diaminobenzidine solution; this was followed by re-staining, alcohol separation, and blocking. Microscopic photography was performed, and the sections were observed using an Olympus CX43 Biological Microscope (Olympus, Japan).

### 2.4 Quantification of αvβ6 and EGFR

For αvβ6 and EGFR protein quantification, IHC slides were digitalized using a Pannoramic DESK, P-MIDI, P250, and P1000 (3DHISTECH, Budapest, Hungary). In the surgical specimens, we separately analyzed tumor and normal tissues. Thereafter, αvβ6 and EGFR were quantified using Image J/FIJI software for at least three fields of view per area of interest (tumor and normal tissue) (20× magnification).

Diaminobenzidine and HE staining were separated using the color deconvolution algorithm, and appropriate threshold levels were set to measure the area of specific diaminobenzidine staining (αvβ6 area) and HE staining (tissue area) to calculate the relative αvβ6 positive area on each image. The thresholds were kept constant within each dataset. We recorded the mean and standard deviation for each sample. For grouped analysis, we used a paired t-test.

### 2.5 Cell Lines and Reagents

Four cell lines were used in this study: CAL27, SCC9, FaDu, and oral normal germ cells (HIOECs). FaDu and CAL27 were procured from Procell Life Science & Technology Co., Ltd. SCC9 cells were purchased from Cobioer Biosciences Co., Ltd. Dulbecco’s modified eagle medium (DMEM) was obtained from Shanghai Zhi Bei Biotechnology Co., Ltd. FaDu cells were cultured in minimum essential medium. CAL27, SCC9, and HIOEC were cultured in DMEM. Cells were cultured in DMEM (Gibco, Invitrogen, Carlsbad, California) supplemented with 10% fetal bovine serum and 1% penicillin/streptomycin at 37°C and 5% ${\mathrm{CO}}_{2}$. To ensure exponential growth, the cells were reseeded weekly.

### 2.6 Mouse Xenograft and Inflammation Models

The School of Pharmacy, Shanghai Ninth People’s Hospital, Shanghai Jiao Tong University approved all animal studies (SH9H-2020-A620-1) and ensured that all procedures were performed in accordance with the National Institutes of Health standards for the care and use of laboratory animals. The mice were maintained on a 12-h light cycle and regularly fed and were divided into three groups (according to the mouse model and the imaging probe), as shown below:

Tongue cancer model: n=6 injections of NHS-ICG, n=6 injections of αvβ6-ICG.

Inflammation model: n=6 NHS-ICG injections, n=6 αvβ6-ICG injections.

Normal mice model: n=6 injected with NHS-ICG, n=6 injected with αvβ6-ICG.

### 2.7 Tumor Xenograft Model

This model included NSG mice (half male and half female; 4 to 6 weeks old). Before xenografting, the cells were washed with PBS and re-suspended in a 1∶1 mixture of PBS and Matrigel matrix (Corning). For *in vivo* and *ex vivo* experiments, we grew *in situ* animal models using 6-week-old nude mice and human tongue squamous cell carcinoma cells (CAL27) by injecting $2\times 10^{7}\;\mathrm{cells}$ in a mixture of culture medium (200  μL). The tumor injectate was infiltrated into the tongues of the mice. When the tumors grew to $50\;{\mathrm{mm}}^{3}$, we performed the experiments after xenografting. The duration of tumor growth was 1 week.

### 2.8 Inflammation Model

This model included BALB/c mice (half male and half female; 4 to 6 weeks old). All BALB/c mice were acclimatized in the laboratory for 4 to 6 weeks, and a 5-mm long, 1-mm deep wound was cut vertically in the midline of the mouse tongue using a scalpel. Live imaging was performed ∼5 to 7 days later.

### 2.9 Reverse Transcription-Polymerase Chain Reaction

Total RNA was extracted from tissues or cells using TRIzol® reagent (Invitrogen; Thermo Fisher Scientific, Inc., Carlsbad, California) according to the manufacturer’s protocols. The extracted RNA was reverse-transcribed using the PrimeScript RT kit with gDNA Eraser (Takara Biomedical Technology Co., Ltd., Beijing, China) according to the manufacturer’s protocols. Quantitative polymerase chain reaction (PCR) was performed using the SYBR Green PCR Master Mix (Takara Biomedical Technology Co., Ltd.) according to the manufacturer’s protocol. Relative expression was normalized to endogenous glyceraldehyde 3-phosphate dehydrogenase (GAPDH). The forward and reverse primer sequences were as follows: AQP9 forward, 5′-AAATAAACCTCCTTGG CCTGA-3′ and reverse, 5′-GCAACAAACATCACCA CACC-3′; E-Cad forward, 5′-CAATGGTGTCCATG GAACA-3′ and reverse, 5′-CCTCCTACCCTCCTGTTCG-3′; α-SMA forward, 5′-TCCCTTGAGAAGAGTTAC GAGTTG-3′ and reverse, 5′-ATGATGCTGTTGTAGGT GGTTTC-3′; N-Cad forward, 5′-CAGTATCCGGTCC GATCTGC-3′ and reverse, 5′-GTCCTGCTCACCACCACTAC-3′; DVL2 forward, 5′-AGTCAGCTCT CATGTTGAGGGT-3′ and reverse, 5′-TACCCAGCCCACACCT TCTT-3′; GSK-3β forward, 5′-CCGACTAACACCA CTGGAAGCT-3′ and reverse, 5′-AGGATGGTAGCCA GAGGTGGAT-3′; CyclinD1 forward, 5′-GAGACCATC CCCCTGACGGC-3′ and reverse, 5′-TCTTCCTCCTCCTCGGCGGC-3′; β-catenin forward, 5′-TGCAGTTCGCCTTCACTATG-3′ and reverse, 5′-ACTAGTCGTGGAATGGCACC-3′; and GAPDH forward, 5′-GCAAGAGCACAAGAGGAAGA-3′ and reverse, 5′-ACTGTGAGGAGGGGAGATTC-3′. Relative expression levels were calculated using the $2^{-\Delta \Delta Cq}$ method.

### 2.10 Western Blot

EGFR and αvβ6 protein expression levels were measured in CAL27, SCC9, FaDu, and HIOEC cell lysates using Western blot (WB) analysis. Proteins were isolated from cells, separated by sodium dodecyl sulfate-polyacrylamide gel electrophoresis, and transferred onto a nitrocellulose membrane. Proteins were detected using antibodies specific for αvβ6 (Abcam ab187155; 1:1000), EGFR (Abcam ab52894; 1:1000), and b-actin (LF202; 1:5000) with a corresponding horseradish peroxidase-conjugated secondary antibody (Abcam ab205718; 1:50,000). An enhanced chemiluminescent substrate (ECL Coloring Solution, Tianneng Company) was used for detection. The αvβ6 and EGFR protein expression levels were measured in clinical tissues using WB analysis as described above.

### 2.11 Histology and Immunofluorescence

The tongues of the mice were harvested and cut into pieces after 1 week, and the tissues were classified according to the type of lesion. One part was fixed in 10% neutral-buffered formalin and embedded in paraffin for IHC analysis. The other part was frozen in an optimal cutting temperature embedding compound, and 30-μm sections were cut for fluorescence imaging using Pannoramic DESK, P-MIDI, P250, and P1000 (3D HISTECH) at the 800-nm channel with 5-μm resolution. For HE staining, serial 5-μm sections were cut.

### 2.12 Confocal Laser Scanning Microscopy

To select a certain type of cell and a suitable probe concentration for the best fluorescence imaging, we performed two experiments. The first experiment was performed using a concentration of 1  μM. We performed the experiment in different cell lines (HIOEC as a normal contrast; the other types were CAL27, FaDu, and SCC9). We pre-inoculated six-well plates with each cell line, treated them with a concentration (1  μM) of fluorescent probes and trypsin-digested cells, and centrifuged them at 300  g for 5 min. We collected the cells and re-suspended $10^{5}$ cells in 200  μL of binding buffer. We added 4  μL of 0.5  mg/mL propidium iodide (PI) and 2  μL of Annexin V-FITC solution. The cells were then incubated in the dark for 15 min at room temperature, away from light. Fluorescence was detected using flow cytometry. The maximum excitation wavelength of Annexin V-FITC was 488 nm, and the emission wavelength was 520 nm. The maximum excitation wavelength of PI was 488 nm, and the emission wavelength was 561nm.

The second experiment was performed using CAL27. We used different concentrations of the fluorescent probes (such as 30, 100, 300, 1000, and 30,000 nM). These steps are similar to those described above.

### 2.13 Cell Viability (CCK8)

The cells were divided into two groups: tumor cell lines (CAL27, FaDu, and SCC9) and a normal cell line (HIOEC). After each type of cell pre-inoculated in 96-well plates was treated with different concentrations of fluorescent probes, 10  μL CCK8 solution (100  μL culture system) was added to each well. Wells with CCK8 and normal cells were used as controls. Cells were incubated in a cell incubator (37°C, 5% ${\mathrm{CO}}_{2}$) for 1 h. The absorbance was measured at 450 nm. The relative viability of the cells in each well was calculated based on the absorbance values.

### 2.14 Data Acquisition and Analysis

Fluorescence was measured in radiant efficiency using Living Image, v4.0 (PerkinElmer, Inc., Waltham, Massachusetts) for Xenogeny-IVIS images and fluorescence counts using Image Studio, v3.0 (LI-COR, Inc., Lincoln, Nebraska) for Odyssey images. Statistical analyses were performed using GraphPad software.

Paired t-tests were used to quantitatively compare fluorescence, except where otherwise noted. Analysis of variance was used to compare fluorescence across several categorical groups (i.e., cell lines). Pearson’s correlation coefficient was used to analyze the relationship between ICG and αvβ6-ICG fluorescence. Statistical significance was defined as p<0.05.

## 3 Results

### 3.1 αvβ6 Is Overexpressed in the Developing Stages of HNSCC

**Fig. 1:**
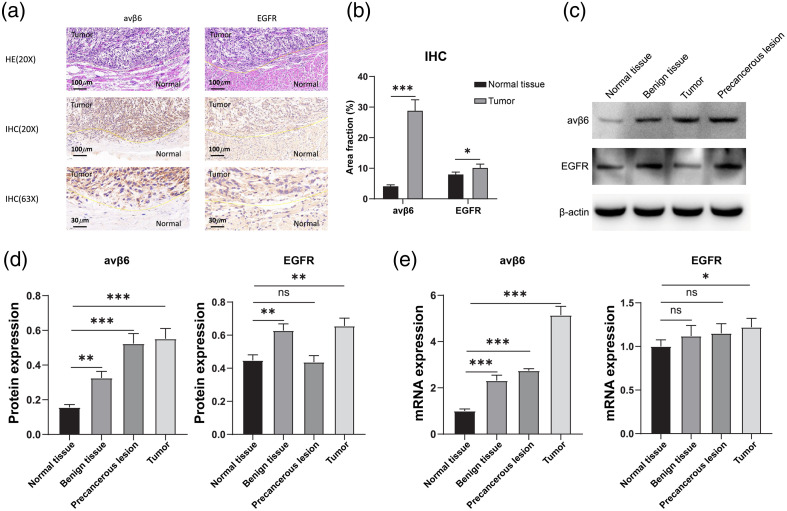
αvβ6 is overexpressed in the developing stages of head and neck squamous cell carcinoma. (a) Paraffin sections of human tissues (n=6) were subjected to HE staining and IHC staining for αvβ6 and EGFR (shooting magnification, 20×, 63×). (b) Quantitative analysis of αvβ6- and EGFR-positive areas. (c) WB detection of αvβ6 and EGFR in tumor tissues with different degrees of malignancy. αvβ6 was expressed in some benign tissues. However, the expression level was lower than that in tumor tissues. (d) Quantitative analysis of αvβ6 and EGFR WB assays (n=6, not significant [ns]: p>0.05, ***: p<0.001, **: p<0.01). (e) Reverse transcription-quantitative PCR detection of αvβ6 and EGFR in tumor tissues with different degrees of malignancy (n=6, ns: p>0.05, *: p<0.05, ***: p<0.001).

To explore the clinical value of an αvβ6-targeted imaging approach in HNSCC, we quantified αvβ6 expression in human surgical biospecimens (n=6). In all specimens, four types of tissue in the developing stages of the disease were analyzed: normal, benign, precancerous lesion, and tumor. Specimens were stained with HE and subjected to IHC. Normal epithelium stained less for αvβ6 than it did for EGFR. In addition, the soft tissue and stroma stained negative for both, allowing clear discrimination of the soft tissue margins [Fig. 1(a)].

Quantification, measured as the αvβ6/EGFR-positive area (protocol described in Sec. 2), revealed a mean value for the two types (αvβ6 versus EGFR) as follows: normal tissue, 4.24±0.40% and 8.25±0.75%; tumor tissue, 28.58±4.18% and 10.25±1.23% (p<0.0001 and p<0.05, respectively) [Fig. 1(b)]. The positive area was proportional to the malignancy of the tumor. The levels of αvβ6 were also examined using WB analysis [Fig. 1(c)].

The quantification methods included PCR-based assays and WB. The result revealed that the expression level of αvβ6 was remarkably upregulated in HNSCC samples compared to that with normal tissues. The differences and amounts of αvβ6 expression were higher than those of EGFR pre- and post-transcription. For micro-RNA expression analysis, the αvβ6 group was as follows: normal=1±0.09; benign=2.31±0.24; precancerous=2.74±0.09; and tumor=5.14±0.38. The EGFR group was as follows: normal=1±0.08; benign=1.12±0.12; precancerous=1.15±0.11; and tumor=1.22±0.10. For WB analysis, the αvβ6 group was as follows: normal=0.16±0.02; benign=0.33±0.04; precancerous=0.52±0.06; and tumor=0.55±0.06. The EGFR group was as follows: normal=0.45±0.03; benign=0.63±0.04; precancerous=0.44±0.04; tumor=0.66±0.05 [Figs. 1(d) and 1(e)].

### 3.2 Characterization of the αvβ6-Targeted Imaging Probe

**Fig. 2:**
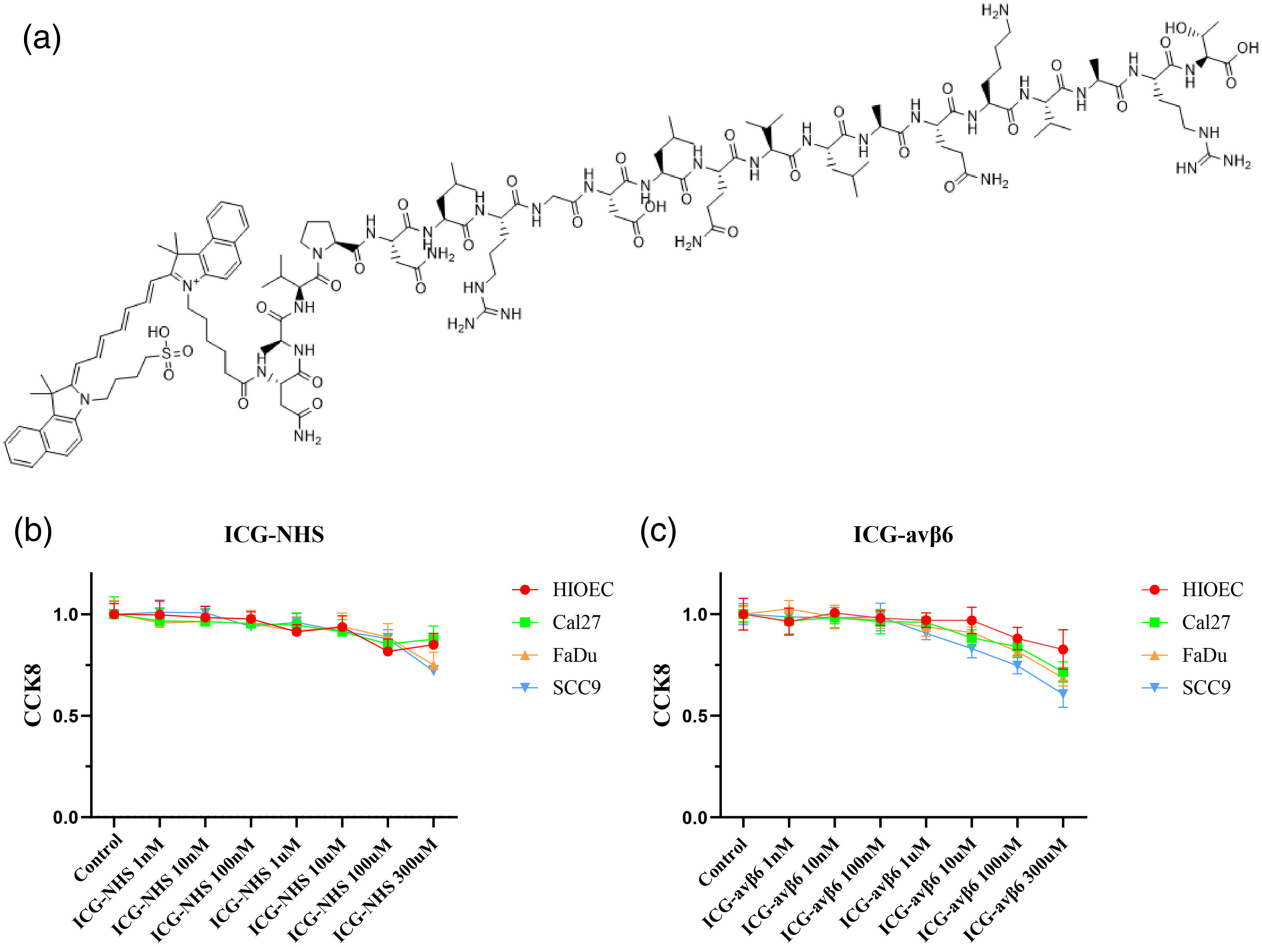
Physicochemical properties and cytotoxicity of the ICG-αvβ6 peptide probe. (a) Sketch of the molecular structure of the ICG-αvβ6 peptide probe. (b) CCK8 toxicity assay in different tongue cancer cell lines with different concentrations of the N-succinimidyl ester (NHS)-ICG fluorescent probe (n=3, set probe concentration gradients: 0.10 nM, 100 nM, 1μM, 10  μM, 100  μM, and 300  μM). (c) CCK8 toxicity assay for different concentrations of the ICG-αvβ6 peptide probe in different tongue cancer cell lines (n=3, set probe concentration gradients: 0.10 nM, 100 nM, 1  μM, 10  μM, 100  μM, and 300  μM).

The peptide selected for probe targeting was delivered using the A20FMDV2 sequence (NAVPNLRGDLQVLAQKVART). ICG was added to the N-terminus of the polypeptide [Fig. 2(a)]. The probe was also analyzed using high-performance liquid chromatography. Therefore, the potential toxicity of the probe is a major concern. Using the CCK8 assay, we found that ICG and the ICG-αvβ6 peptide probe had extremely low toxicity. Toxicity mainly depended on the concentration of the probe, as shown in Figs. 2(b) and 2(c). The results showed that a concentration <1  μM did not influence cell growth. The HIOEC cell line was used as a control [Figs. 2(b) and 2(c)].

### 3.3 Validation of Probes for *In Vitro* Imaging

The ability of the probe to identify high and low αvβ6-expressing tumors was assessed in three kinds of HNSCC cells (CAL27, SCC9, and FaDu) using HIOEC cells as the control.

**Fig. 3:**
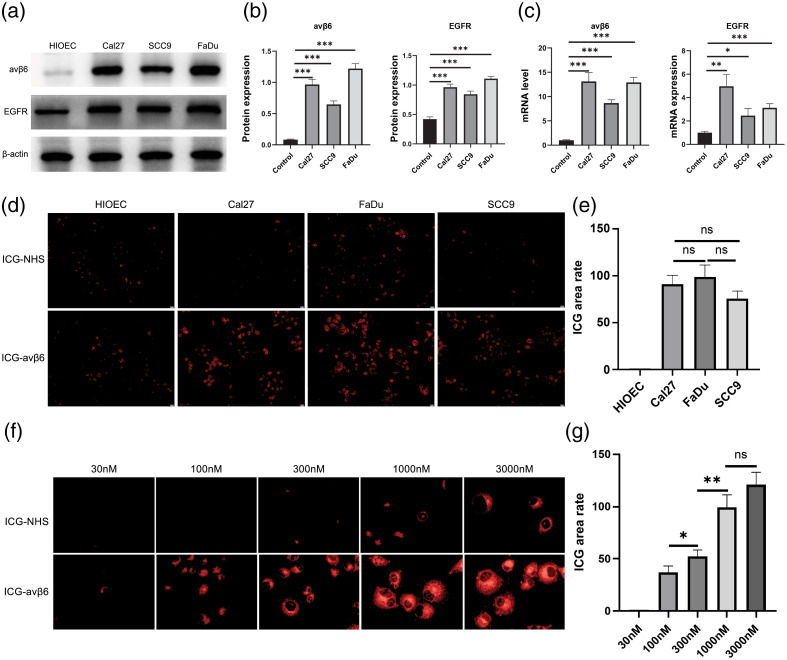
Validation of probes for *in vitro* imaging. (a) WB detection of αvβ6 and EGFR in different cell lines. (b) Quantitative analysis of αvβ6 and EGFR WB assays in different cell lines (n=3, ***: p<0.001). (c) Reverse transcription-quantitative PCR detection of αvβ6 and EGFR in different cell lines (n=3, *: p<0.05, **: p<0.01, and ***: p<0.001). (d) ICG and the ICG-αvβ6 peptide probe were incubated in different cell lines for laser confocal microscopy imaging (probe concentration set to 1  μM, microscopy magnification 10×). (e) Fluorescence intensity statistics of different cell lines. (f) Laser confocal microscopy imaging of CAL27 incubated with different concentrations of N-succinimidyl ester (NHS)-ICG and ICG-αvβ6 peptide probe (set probe concentration gradients: 30 nM, 100 nM, 300 nM, 1  μM, 3  μM; microscopy magnification 40×). (g) Fluorescence intensity statistics of different probe concentrations of CAL27 (not significant [ns]: p>0.05, *: p<0.05, and **: p<0.01).

αvβ6 and EGFR levels in the cells were verified using WB [Fig. 3(a)]. Densitometric quantification of the blots showed FaDu>CAL27>SCC9>control [Fig. 3(b)]. Densitometric quantitation of the quantitative polymerase chain reaction (PCR) showed that FaDu=CAL27>SCC9>control [Fig. 3(c)]. To determine the selective cell binding and uptake of the probe, cells were incubated with it for 30 min. This was corroborated using confocal microscopy, where the cellular accumulation of ICG and the ICG-targeting αvβ6 fluorescent imaging agent could be clearly seen [Fig. 3(d)], and fluorescence intensity quantification showed that HIOEC<SCC9<CAL27<FaDu [Fig. 3(e)].

The ICG-targeting αvβ6 fluorescent agent showed a better imaging effect than did ICG. The quantitative metric was “fluorescence intensity,” as shown in Fig. 3(g). Based on the growth rate and fluorescence intensity, CAL27 was selected as the experimental model to identify the suitable concentration for fluorescence imaging. Optimal probe concentration was determined by evaluating different concentrations: 30 nM, 100 nM, 300 nM, 1  μM, and 3  μM [Fig. 3(f)]. The mean fluorescence intensity quantification showed an increasing trend below 1  μM. However, there was no difference in imaging when using 3 or 1  μM [Fig. 3(g)].

### 3.4 ICG Targeting αvβ6 Fluorescent Probe Accumulates in Tumor Tissues and Identifies Margins

The animals were divided into three groups: tumor, inflammation, and normal (n=6 each). Two animal models were created for this experiment.

**Fig. 4:**
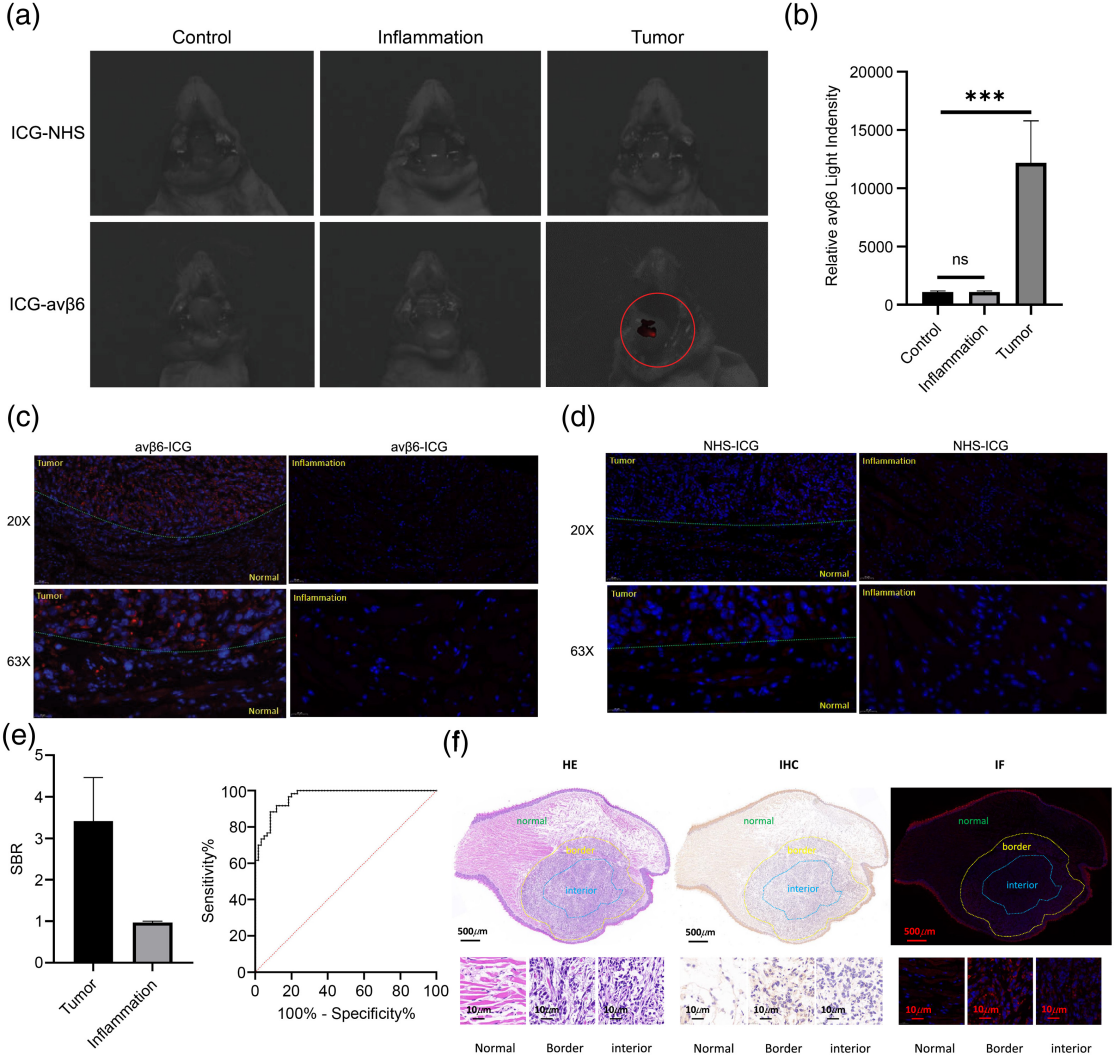
*In vivo* imaging and histological analysis with fluorescent probes. (a) IVIS assay of the animal model through vein injection of the fluorescent probe (n=6, injection concentration: 100  μM, injection dose: 100  μL). (b) Relative αvβ6 light intensity ($p/s/{\mathrm{cm}}^{2}/\mathrm{sr}\mu w/{\mathrm{cm}}^{2}$) (ns, not significant). (c) Frozen sections of probe-labeled tumor tissue were examined using IF (n=6, section thickness 5  μm, microscopy magnification 20×, 63×). (d) IF detection of the probe in frozen sections of inflammation tissue (n=6, section thickness 5  μm, microscopy magnification 20×, 63×). (e) SBRs of the ICG-αvβ6 peptide probe. Receiver operating characteristic curves for the whole tumor. (f) *In vivo* labeling of frozen sections of tumor tissue for HE, IHC, and IF detection (section thickness 5  μm, microscopic magnification 2×, 63×).

Tumor models were established on the tongues of NSG mice. Tumor growth was evident after an incubation period of 7 days for the CAL27 xenografts and was recorded and observed after injection. After the tumor diameter had increased to nearly $50\;{\mathrm{mm}}^{3}$, the mice were euthanized, and fluorescence imaging was subsequently performed. An inflammation model was established concomitantly with the tumor group. Mice were imaged 24 h after injection with the αvβ6-targeted fluorescent imaging agent. The probe was administered intravenously, and tumors were imaged using an IVIS epifluorescence-injected imaging system. We identified the αvβ6-targeted imaging probe in the areas of the tongue where tumors were present, whereas a lower signal was observed in the surrounding normal tissues and control tongues [Fig. 4(a)].

In the αvβ6-targeted probe imaging group, the average fluorescence signal in the tumor area was 12 times higher than that in the other two groups. The signals were similar between the inflammatory and normal groups.

It is clinically difficult to distinguish between inflammation and tumors with the naked eye; therefore, we established an inflammation model to verify the probe’s ability to distinguish between the two different types. Control and inflamed tongues had significantly lower relative radiance efficiency signals (p<0.001) than xenografted tongues [Fig. 4(b)].

### 3.5 αvβ6-Targeted Imaging Probe Accumulation in Margins and Sensitivity/Specificity Analysis

We analyzed the *in situ* diagnostic performance of αvβ6-targeted fluorescent imaging agent tumor uptake using the immunofluorescence (IF) results of the slices. We observed that the tumor area had higher fluorescence intensity than the normal area (20× and 63× magnification) in the αvβ6-ICG group. However, the ICG group showed little difference in fluorescence intensity between the tumor area and the tissue adjacent to cancer cells [Fig. 4(c)].

The signal-to-background ratio (SBR) was obtained by dividing the fluorescence intensity of the selected region by the average intensity of the normal tissue region. Inflammatory tissue fluorescence intensity was low and showed little difference between the two probes [Fig. 4(d)].

The SBR was 2.94±1.13 standard deviation in the tumor group and 0.97±0.03 standard deviation in the inflammation group [Fig. 4(e)].

A receiver operating characteristic curve was constructed, which demonstrated an area under the curve of 0.965 with a SBR of 1.52, corresponding to a sensitivity of 91.67% and specificity of 88.33% [Fig. 4(f)].

The αvβ6-targeted peptide probe was most intense at the periphery of tumors. The dotted line divides the tumor into three parts: normal, border, and tumor. Confocal microscopy images of tumor samples showed abundant probes in αvβ6-expressing tumor cells. The border of the tumor had higher fluorescence intensity than the interior of the tumor. In benign samples, αvβ6 staining was confined to the thin αvβ6-expressing basal layer of the epithelium [Fig. 4(f)].

## 4 Discussion

In this study, we evaluated the ability of αvβ6-ICG to accurately identify the HNSCC. IHC staining in human specimens showed that the expression of αvβ6 increased with increasing malignancy grade of cancer. In addition, at the cellular level, the expression of this target was higher in HNSCC than in normal cells. Confocal microscopy imaging of cells also revealed that the probe was localized in the cytoplasm. We selected the CAL27 cell line with high αvβ6 expression and rapid proliferation to construct an *in situ* tongue cancer model. The probe showed tumor-specific uptake. Furthermore, compared with the mouse tongue inflammation model, no probe uptake was observed. Therefore, we concluded that the probe could specifically identify tumors and distinguish inflammatory tissues.

In the *in situ* analysis of the fluorescence of tissue sections, we performed HE and IHC staining of the pathological sections. In our *in situ* analysis of the fluorescence of tissue sections, the probe showed high sensitivity and specific uptake in the tumor area. The high concentration observed at the tumor periphery indicates that the probe could outline the distinguishing edges well.

The treatment for HNSCC is curative surgery, which aims to completely remove the local or locoregional disease to achieve a long-term disease-free status. In addition to this fundamental basis of surgical resection, the preservation of surrounding normal tissues is closely related to the quality of life.^27^ When tumors are operable, the initial treatment involves wide excision with adequate margins.^28^ In addition, the complete removal of clinically overt lesions, potentially malignant (premalignant) lesions, or undetectable (microscopic) disease is a crucial factor. However, *ex vivo* histopathological assessment of paraffin-embedded sections remains the gold standard. During the operation, the specimens were subjected to freezing analysis. However, these methods have limitations, such as specialization of surgeons,^29^ long waiting time,^30^ high cost, invasive tests,^31^ and deformation of specimens.^32^ These factors significantly influence the accuracy of margin resection. According to the current treatment guidelines of the National Comprehensive Cancer Network Clinical Practice Guidelines in Oncology,^33^ the criterion for clear resection margins is 5 mm, regardless of primary tumor size or characteristics.

In recent years, NIR has emerged as a promising tool for analytical applications, particularly *in vivo* imaging and therapy. Multiple contrast agents have been tested in clinical trials to assist doctors in HNSCC surgery. These include cetuximab-800CW,^34^ panitumumab-IRDye800CW,^35^ EMI-137,^7^ PARPi-FL,^36^ and ICG.^36^ Some of these are nanoprobes, such as ICG-Nanocoll,^37^ ICG-(99m) Tc-nanocolloid hybrid tracer, and (99m) Tc-nanocolloid.^38^ Some are non-fluorescent dyes, such as Lugol’s iodine 31^39^ and toluidine blue 32.^40^

αvβ6-targeted peptide efficiently targets tumor cells within the cytoplasm and cell membrane. The structure comprises two parts, ICG and the αvβ6 peptide. Integrin αvβ6, the only integrin with epithelial specificity, is hardly expressed in normal epithelium. Its high expression in many malignant epithelial tumors plays an essential role, making it a promising target for diagnosis and treatment. R01-MG-IRDye800 has been used to treat pancreatic ductal cancer.^41^ IRDye800-PEG28-A20FMDV2-K16R-PEG28 was used to image an orthotopic pancreatic tumor model.^42^ This target has been used to treat breast, colon, lung, and pancreatic cancers. These novel integrin αvβ6-targeted NIR fluorescence dyes showed notable imaging effects, which elevated contrast and uptake during the imaging. However, for HNSCC, targeted QD-A20 is selective to cells overexpressing αvβ6 integrin.^43^ Considering the safety of probe use, nanoprobes have rarely been used in clinical experiments. To bridge this gap, we designed a small molecule probe targeting αvβ6 with low toxicity.

EGFR is the most targeted protein in HNSCC. The IHC of human tissues showed that αvβ6 staining in tumors is stronger than that of EGFR and results in better differentiation from normal tissues. αvβ6 expression in tumors and normal tissues differed more than that of EGFR. Moreover, αvβ6 expression showed a better incremental relationship with tumor malignancy than did EGFR.

The aim of this study was to develop an optical probe for *in vivo* imaging while determining the intracellular location of the αvβ6 peptide. Confocal microscopy was used to assess whether the peptide was present in the cytoplasm or cell membrane. *In vitro* and *in vivo* evaluation of αvβ6 targeting was performed in cell lines with different levels of αvβ6 integrin expression; CAL27, FaDu, and SCC9 were selected as αvβ6-positive cell lines and HIOEC as αvβ6-negative. The probes showed an affinity for αvβ6-positive cell lines and no binding to αvβ6-negative cell lines, confirming the selective retention of αvβ6. In addition, the fluorescence intensity showed a dose-escalating correlation.

We used whole mouse tissue sections to confirm that the αvβ6 peptide aggregates in tumor regions and has a lower signal in normal and inflammatory tissues. Therefore, it is possible to effectively distinguish between inflammation and tumors. In our *in situ* analysis of the fluorescence of tissue sections, αvβ6 showed high sensitivity and specific uptake in the tumor periphery. IHC showed that αvβ6 staining was less prominent within the tumor core than in the surrounding tissues. From a surgical perspective, enhancement of the tumor periphery might be advantageous. This is because the challenge of complete tumor resection is to identify residual lesions at the tumor margins.

The rapid uptake and internalization of the probe suggest that from a clinical translation perspective, αvβ6-based probes can be administered several hours before imaging, providing a significant advantage over EGFR antibody-based probes administered several days before surgery. Visualizing tumors intraoperatively using NIR fluorescence agents allows for more precise resection and improves safety by avoiding unnecessary damage to normal tissues with no known toxic effects.

## 5 Conclusions

We demonstrated specific and sensitive uptake of the αvβ6-targeting imaging probe in the *in situ* tumor model of HNSCC. In addition, biomarker expression increased as the malignancy grade increased. This probe may play an essential role in early screening and intraoperative fluorescence imaging of HNSCC.

## Data Availability

Data sharing is not applicable to this article, as no new data were created or analyzed.
